# Virome Analysis and Association of Positive Coxsackievirus B Serology during Pregnancy with Congenital Heart Disease

**DOI:** 10.3390/microorganisms11020262

**Published:** 2023-01-19

**Authors:** Mathieu Garand, Susie S. Y. Huang, Lisa S. Goessling, Fei Wan, Donna A. Santillan, Mark K. Santillan, Anoop Brar, Todd N. Wylie, Kristine M. Wylie, Pirooz Eghtesady

**Affiliations:** 1 Division of Pediatric Cardiothoracic Surgery, Department of Surgery, Washington University School of Medicine, St. Louis, MO 63110, USA; 2 Department of Public Health Sciences and Surgery, Washington University School of Medicine, St. Louis, MO 63110, USA; 3 Department of Obstetrics and Gynecology, University of Iowa, Iowa City, IA 52242, USA; 4 Department of Pediatrics, Washington University School of Medicine, St. Louis, MO 63110, USA

**Keywords:** enterovirus, coxsackievirus B, cardiotropic virus, viremia, anti-CVB IgG/IgM, maternal virome, pregnancy

## Abstract

Background: We have previously shown coxsackievirus B (CVB) to be a potent inducer of congenital heart disease (CHD) in mice. The clinical relevance of these findings in humans and the roles of other viruses in the pathogenesis of CHD remain unknown. Methods: We obtained plasma samples, collected at all trimesters, from 89 subjects (104 pregnancies), 73 healthy controls (88 pregnancies), and 16 with CHD–affected birth (16 pregnancies), from the Perinatal Family Tissue Bank (PFTB). We performed CVB IgG/IgM serological assays on plasma. We also used ViroCap sequencing and PCR to test for viral nucleic acid in plasma, circulating leukocytes from the buffy coat, and in the media of a co-culture system. Results: CVB IgG/IgM results indicated that prior exposure was 7.8 times more common in the CHD group (95% CI, 1.14–54.24, adj. *p*-value = 0.036). However, the CVB viral genome was not detected in plasma, buffy coat, or co-culture supernatant by molecular assays, although other viruses were detected. Conclusion: Detection of viral nucleic acid in plasma was infrequent and specifically no CVB genome was detected. However, serology demonstrated that prior CVB exposure is higher in CHD-affected pregnancies. Further studies are warranted to understand the magnitude of the contribution of the maternal blood virome to the pathogenesis of CHD.

## 1. Introduction

Every 30 minutes, nearly 80 babies are born with congenital heart disease (CHD) world-wide [1], representing 1:100 births. In these children, CHD is an important source of mortality and morbidity. The causes of the disease remain elusive, although genetic factors appear to play an important role, particularly in concert with environmental factors. Among the latter, one of the oldest and best recognized pertains to maternal viral infections, as first highlighted during the 1960 Rubella epidemic. A study published in 1972 by Gordon and Karunas investigated 22,935 women and revealed a significant association between antibody titers for CVB3 and CVB4 and cardiovascular anomalies. With a greater occurrence of CVB3 and CVB4 infections in mothers of infants with anomalies compared to controls (10% vs. 2% with *p* < 0.01, and 19% vs. 11% with *p* < 0.05, respectively) [2]. When considering infection with any serotypes of CVB, the occurrence was significantly greater in women who gave birth to babies with cardiovascular anomalies compared to healthy controls (36.7% vs. 26.7% with *p* < 0.05). More recently, a meta-analysis encompassing 17 studies showed that first trimester maternal viral infections are significantly associated with risk of CHD, even in the current era [3].

Using an animal model, our group has found that maternal coxsackievirus infection induces heart defects in mice [4]. *Enteroviruses B* (EVB) species are particularly well known for their ability to infect cardiomyocytes and are a significant cause of neonatal and pediatric myocarditis [5]. Indeed, EVB can access and injure the fetus through its cognate receptors, the Coxsackievirus and Adenovirus Receptors (CARs), which are expressed on the surface of the fetal cardiomyocytes, human placenta, and fetal brain [6]. Additionally, the neonatal Fc receptor which is expressed in the placenta to facilitate the transport of maternal IgG to the fetus, was recently reported to be exploited by a large group of EVB for cell entry [7]. CAR itself is essential, as knockouts in mice lead to severe CHD and embryonic lethality [8]. In our murine model, we found in utero exposure to Coxsackievirus B (CVB), a group of strains of the EVB species, leads to upwards of 30% incidence of CHD, particularly ventricular septal defects (VSD) [9]. Furthermore, we have shown from examination of clinical data and second and third trimester blood samples, that pulmonary valve abnormalities and VSD are associated with possible exposure to CVB during earlier time points in pregnancy [10,11]. These observations led us to question the magnitude of linkage between CVB infections and the occurrence of CHD. The evidence accumulated so far on the contribution of viruses to the pathogenesis of CHD has been limited to a small number of viruses [3,12].

Much less is known about other viruses, partly due to technical challenges. Diagnostic methods typically include molecular assays, serology, histology, and occasionally culture. Recently, metagenomic sequencing has proved to be a powerful tool for viral pathogen detection [13,14]. The advantages are that no a priori knowledge of which viruses might be present is needed, the results provide a comprehensive assessment of all viruses simultaneously, and viruses with highly divergent genomes can be detected [15]. Conversely, challenges in blood virome analyses have been identified with direct metagenomic sequencing approaches, including the major sensitivity “interference” created by the larger human genome present in the samples and detection of bacterial genomes [16]. ViroCap is a panel of short biotinylated probes that target the complete genomes of all known vertebrate viruses. The probes can be hybridized to target genomic material in a sample and enriched by streptavidin capture of the biotin. This greatly improves viral detection in clinical samples [15,17]. Careful classification of sequence reads is required to identify viral sequences and minimize false positives. For this, genome analysis should include quality control in enriched samples to appropriately assigned non-viral contamination. For example, using ViroMatch [18], an optimized viral sequence aligner, only sequences with an unambiguous mapping to a viral reference are counted as viral hits. 

The Perinatal Family Tissue Bank (PFTB) at the University of Iowa is a longstanding biobank collecting biological samples with extensive clinical data [19]. It provides access to serial samples of blood collected shortly after diagnosis of pregnancy through the final outcome of pregnancy. This is a unique cohort where blood samples are obtained early on in pregnancy prior to any feasible diagnosis of CHD which typically occurs in the second trimester. The objectives of the current study were to determine the anti CVB IgG/IgM antibody titers in the plasma of pregnant women during pregnancy using by clinical-grade serology assays, to determine the presence of viral nucleic acid in plasma and peripheral blood lymphocytes using ViroCap, and to assess the association between CVB burden and CHD outcomes. Using ViroCap [15,17], we detected several viruses in the plasma, however we did not directly detect the CVB genome. Overall, our findings provide an overview of viral exposure in pregnancies complicated by CHD and provide further evidence in support of the potential role of CVB in the pathogenesis of CHD.

## 2. Materials and Methods

### 2.1. Subject Recruitment and Sample Collection

Plasma samples are collected longitudinally from women throughout their pregnancies via the PFTB program at the University of Iowa [19]. In brief, maternal blood is collected following informed consent. All pregnancies are followed to the final determination of pregnancy outcome and allow for the querying of the past and future medical records from consented participants, and for the children until they turn 18 (IRB# 200910784). The stages of pregnancy were defined as follows: 1st trimester—conception to 12 6/7 weeks, 2nd trimester—13 0/7 to 24 6/7 weeks, and 3rd trimester—greater than 25 0/7 weeks. After review of the ICD-10 codes and confirmation by examination of echocardiograms by the senior author, a pediatric cardiovascular surgeon (PE), and a maternal fetal medicine subspecialist (MKS), 16 pregnancies (from 16 women) were found to result in a child affected with CHD. A cohort of 88 healthy pregnancies (from 73 women) from whom at least one first trimester plasma sample was available constituted the Controls group, representing a healthy to CHD-affected pregnancy ratio of 5.5 to 1. If available, concentrated leukocyte samples (buffy coat) corresponding to these plasma samples were obtained. All samples were stored at −80 °C until analysis.

### 2.2. Diagnosis of Congenital Heart Disease

To categorize the CHD groups, we reviewed neonatal echocardiography reports to confirm the initial diagnoses based on ICD-10 codes assigned during collection of paired maternal–child samples into PFTB. CHD was further categorized according to the following: right-sided lesions, left-sided lesions, conotruncal defects, laterality defects, isolated septal defects (including atrioventricular), and complex defects/other groups. 

### 2.3. CVB Serology

Plasma samples were analyzed for the presence of anti-CVB (B1, B3, and B5) antibodies using the Serion enzyme-linked immunosorbent assay (ELISA) kits (ESR134M and ESR134G; QED Biosciences, Inc., San Diego, CA, USA). According to the manufacturer’s guidelines, antibody titers (anti-viral IgG or IgM antibodies) greater than 15 U/mL are designated as positive or elevated, titers between 11 and 15 U/mL are considered borderline, and titers below 11 U/mL are designated as negative. We defined seroconversion as a change from negative to positive titers when comparing the early time point (1st trimester) with later time points (2nd and 3rd trimesters).

### 2.4. Nucleic Acid Isolation

Viral nucleic acid was isolated using the Quick DNA/RNA Viral Kit (Zymo Research, catalog# D7020) according to the manufacturer’s instructions. For buffy coat cells, approximately 2 × 10^6^ cells were washed briefly with 1.5 mL Hanks Balanced Salt (HBSS, Corning #21-022-CV) solution to remove dimethyl sulfoxide (DMSO). Washed cells were pelleted by centrifugation (400× *g*, 10 min), the supernatant removed, and the pellet resuspended in 200 μL HBSS. Viral nucleic acid was extracted with the High Pure Viral Nucleic Acid Kit (Roche, Basel, Switzerland, catalog #11858874001) according to the manufacturer’s instructions. For co-culture experiments, the media was concentrated 10-fold (2 mL to 200 µL) with an Amicon Ultra-4 centrifugal filter unit (Millipore-Sigma #UFC80500) as per the manufacturer’s instructions. Nucleic acid was isolated from the concentrated sample with the High Pure Viral Nucleic Acid Kit (Roche) according to the manufacturer’s instructions. All nucleic acid samples were stored at −80 °C.

### 2.5. Buffy Coat Co-Culture Experiments and Specific Viral Detection by PCR

Co-culture of buffy coat leukocytes with mammalian cells was performed using recovered frozen buffy coat cells. Four cell lines, all purchased from ATCC, were mixed equally, and used for the co-culture: Buffalo Green Monkey Kidney cells (BGMK-DAF, PTA-4594), Colorectal Adenocarcinoma cells (Caco-2, HTB-37), HeLa-derived Epithelial Carcinoma cells (HEp2, CCL-23), and lung fibroblast cells (MCR-5, CCL-171). The day prior to co-culture, a T25 flask was seeded with 2.5 × 10^6^ cells from each line in complete media consisting of Minimal Essential Media (MEM, Corning #10-009-CV) containing 10% fetal bovine serum (FBS, Gibco #26140-079) and 1X GlutaMAX (Gibco #35050-061). The following day, the complete media was removed and replaced with 2 mL co-culture media which has the same components as complete media but with a lower FBS concentration (5%) and the addition of Pen/Strep solution to 1X (100X solution, Gibco #15140-122). Frozen buffy coat cells were quick thawed by brief immersion in a 37 °C water bath, mixed by inversion and 100 μL (approximately 1 × 10^6^ cells) was added to 600 μL of complete media to wash out the DMSO. Cells were pelleted at 400× *g* for 10 min, resuspended in 1 mL co-culture media, and added to the semi-confluent T25 flask of mammalian cells. Cells underwent three serial passages over 13 days and were examined daily for signs of cytopathic effect. On the last day of culture, flasks were subjected to three freeze/thaw cycles to lyse cells and release virus into the culture media. The media, approximately 3 mL, was collected, centrifuged to remove cell debris (2800× *g* for 10 min), and the clarified media stored at −80 °C.

All PCR reactions performed with nucleic acids extracted from buffy coat and media from the co-culture system were conducted in a total volume of 25 μL containing 0.2 μM of each primer and 3 μL of nucleic acid. RT-PCR to detect Enteroviruses was performed with SuperScript III One-Step RT-PCR System with Platinum Taq DNA polymerase (Invitrogen #12574) with the following cycling conditions; 55 °C for 30 min, one cycle of 94 °C for 5 min followed by 40 cycles of 94 °C for 20 s, 58 °C for 30 s, 68 °C for 30 s, followed by a hold cycle at 4 °C. PCR to detect the other viruses was performed with Platinum II Hot-Start Green PCR Master Mix (Invitrogen #14001) with the same cycling conditions as for RT-PCR excluding the initial 30 min, 55 °C incubation. PCR amplicons were analyzed on a 1.8% agarose gel containing 1X SYBR Safe Green (Invitrogen #S33102). Virus positive and negative (water) controls were run with each reaction. Controls containing inactivated virus in a matrix designed to mimic clinical specimens were purchased to validate the nucleic acid isolation and RT-PCR and PCR reactions. The following controls were purchased from ZeptoMetrix NATtrol controls: Cytomegalovirus (CMV) strain AD-169 (NATCMV-0002), Enterovirus EV Panel (NATEVP-C), Epstein–Barr virus (EBV) strain B95-8 (NATEBV-0003), Adenovirus Low Run Control (ADVL101), and Human Herpesvirus-6 (HV6L101). Primers used for RT-PCR/PCR are shown in Table S1 (Supplementary Materials).

### 2.6. Viral Sequence Detection by ViroCap and Sequence Data Analysis

ViroCap targeted sequence capture and sequencing were performed on total nucleic acid. ViroCap probes are used after library construction to enrich viral nucleic acids as described in [15,17]. In brief, for the plasma, total nucleic acid was pooled (*n* = 4–5 samples per pool) prior to sequence library construction. For the buffy coat samples, each sample was processed independently. The RNA in the total nucleic acid was reverse transcribed with random primers, and the resulting cDNA and DNA were used as input for dual-indexed libraries constructed with the Swift Biosciences Accel NGS Kit. Libraries were pooled and mixed with ViroCap targeted sequence capture probes (synthesized by Roche NimbleGen, Madison, WI, USA) to enrich viral targets according to the manufacturer’s instructions [17]. Enriched libraries were sequenced on the Illumina NovaSeq platform (Illumina, San Diego, CA, USA). Virome sequence analysis was carried out using ViroMatch software [18] which employs nucleotide and translated amino acid sequence alignments to allow for the identification of both conserved and divergent viral sequences. In brief, sequences are trimmed to remove sequencing adaptors and low-quality regions. Low complexity sequences are masked. Human sequences are identify using BWA-MEM and removed. Non-human sequences are subject to nucleotide sequence alignment with BWA-MEM and translated alignment with Diamond against databases containing all viral genomes. These putative viral sequences are then re-aligned to the comprehensive NCBI databases using BWA-MEM and Diamond to confirm viral positivity. Sequences are only considered viral if they are unambiguously assigned virus classification. By default, classification is reported at the genus level. Species level classifications are manually reviewed to confirm accurate assignment. For the buffy coat samples that were sequenced, contaminating viruses were identified and removed from analysis: bovine viral sequences, likely from serum, and SARS-CoV-2 from viral screening that was underway at the sequencing center during that time.

### 2.7. Data Analysis

Relevant clinical parameters were compared between the Controls and CHD groups using Wilcoxon Rank Sum Test or Chi square as appropriate, with significance denoted at *p* < 0.05. For the CVB serology analysis, positive or elevated titers were determined independently for both IgG and/or IgM as per the manufacturer’s guidelines. IgM is produced in the early stages of infection, and IgG is produced later; thus, we measured both in order to encompass a wider range of immune responses. A positive ‘call’ for anti-CVB antibodies was defined as having either elevated/positive IgG or IgM titers, or both. We used the Fisher Exact Test with 3 conditions to assess any statistically significant differences between the proportion of positive titers among Controls and CHD groups. We then used logistic regression to determine the odds ratio of CVB positivity in CHD vs. Controls, while adjusting for (i) Subject (repeated measures) as random effects, and (ii) GDM (confounder) as fixed effects; we assigned borderline titers results to negative.

## 3. Results

### 3.1. Cohort Description

**Figure 1:**
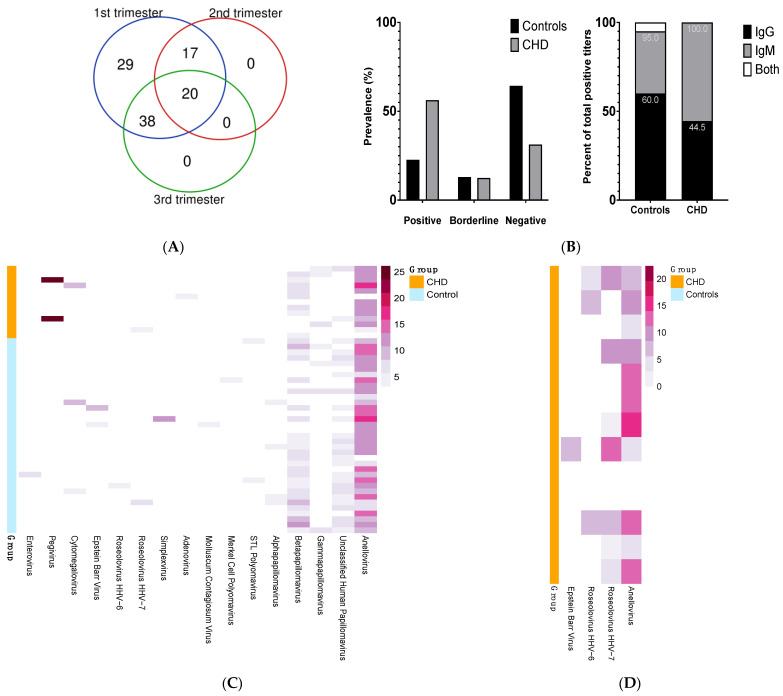
(**A**) Distribution of samples collected by trimester for all pregnancies (total = 104). (**B**) Prevalence of CVB serology calls among Control and CHD groups (top histogram) and proportion of IgG and IgM serology results among individuals identified as positive for CVB (bottom stacked bars). (**C**) Distribution of viruses detected by ViroCap among the pools of CHD-affected (CHD) and healthy controls (Controls) pregnancies. Each row represents a pooled sequencing library (*n* = 4 or 5 plasma samples per pool). Plasma samples were collected at various times through pregnancy (weeks 5–41 of gestational age). Columns show virus detected (the gradient color of the bar correlates to the log2 normalized sequencing reads). (**D**) Distribution of viruses detected in the buffy coat by ViroCap in a subset of individuals. Each row represents individual library. Buffy coat samples were collected at 1st trimester visit. Columns show virus detected (the gradient color of the bar correlates to the log2 normalized sequencing reads).

A total of 220 plasma samples were collected during the 104 pregnancies of 89 women. The distribution of samples collected by trimester is summarized in Figure 1A. The 89 pregnant women were partitioned between healthy (Controls, *n* = 73) and CHD-affected (CHD, *n* = 16). The major demographic was White and non-Hispanic or Latino. Among the Controls, there were 88 pregnancies, i.e., some women were enrolled in the study across multiple pregnancies. Only gestational diabetes mellitus was significantly different between the Controls and CHD groups (0% vs. 18.75%, *p* < 0.01). Additional cohort characteristics are presented in Table 1. The different CHD diagnoses were as follow: ventricular septal defects or VSD (*n* = 7), coarctation/VSD (*n* = 2), pulmonary valve stenosis (*n* = 2), atrial septal defects or ASD (*n* = 1), ASD/mitral valve regurgitation (*n* = 1), coronary artery fistula (*n* = 1), and truncus arteriosis (*n* = 1).

### 3.2. CVB Serology Indicated Increase Exposure/Prevalence of Anti-CVB Antibodies in the CHD Group

We assayed plasma samples from all trimesters, placing emphasis on having at least one first trimester sample for each subject; if more than one sample was collected during the first trimester for a subject, we selected the sample associated with the earliest gestational age. In order to encompass multiple stages of infection, we determined titers for both IgG and IgM. In total, 27% of the women assayed had evidence of prior or early exposure to CVB (Supplementary Table S2). IgM serology result, but not IgG, was significantly different between Controls and CHD (odd ratio of 4, 95% CI, 1–16, adj. *p*-value = 0.049). The prevalence of positive serology (i.e., positive ‘call’, see Materials and Methods section) was 56.25% and 22.73% in CHD vs. Controls, respectively (adj. *p*-value = 0.02) (Figure 1B, histogram). Of the pregnancies in the Controls group (*n* = 88), 22.72% (*n* = 20) had positive CVB titers and 13.63% (*n* = 12) had borderline titers. In contrast, in the CHD group (CHD, *n* = 16), 56.25% (*n* = 9) had positive CVB titers and 12.50% (*n* = 2) were borderline in value. Titer results were not associated with gestational age at time of sampling. Using logistic regression, the serology results indicated that prior CVB exposure was 7.8 times more common in the CHD group compared with the Controls (95% CI, 1.14–54.24, adj. *p*-value = 0.036). Among Controls identified as positive, 60%, 35%, and 5% correspond to IgG, IgM, and both IgG/IgM results, respectively. In the CHD group, 44.5% and 55.5% of positive results correspond to IgG and IgM, respectively (Figure 1B, stacked bars).

In our cohort, there were three women with multiple pregnancies and we noted the following serological observations. One had elevated IgG titers in the first pregnancy which was complicated by CHD (VSD) and was found negative 12 months later during an uncomplicated second pregnancy. A second subject had first trimester IgM titers seroconversion during the first uncomplicated pregnancy which remained high during her second pregnancy, 4 years later, that was complicated by CHD (VSD). The third subject had positive IgM titer in the first trimester of her first pregnancy which was complicated by CHD (VSD), but the titers reverted to negative from the second trimester onward and for the second uncomplicated pregnancy 2 years later.

### 3.3. Detection of Viral Nucleic Acid in Plasma during Pregnancy

ViroCap was performed in pools of 4–5 plasma samples with 13 pools for CHD, and 35 for Controls (Table S3). The distribution of the viruses detected are presented in Figure 1C. Evidence of at least one pathogenic virus in the maternal circulation was found in 17% of the Controls and 38% of the CHD pools. Of note, CVB was not detected in any pool. Viral sequences from 14 and 8 distinct viruses were identified in the Controls and CHD groups, respectively. Both groups of mothers had common vertebrate-infecting viruses such as anelloviridae, members of the papillomavirus family, cytomegalovirus (CMV), and roseolovirus (HHV-6B and HHV-7). Of note, the distributions of anellovirus and betapapillomavirus were nearly ubiquitous and similar across the groups. These viruses were not subtyped, as we focused on the other viral types, which are more likely to be pathogenic. Viruses only found in plasma of the CHD group were pegivirus and adenovirus. Inversely, enteroviruses (i.e., rhinovirus A) were unique to control pools.

### 3.4. Detection of Viruses in Buffy Coat and Co-Culture System

We explored the feasibility of using buffy coat samples to detect viral infection. Enterovirus B serotypes such as coxsackievirus B can transform into a non-cytolytic form [20,21], enabling persistence of the virus in human tissues [22]. As compared to direct assays of plasma or leukocytes, Toniolo et al. showed that enterovirus detection rate can be enhanced by co-culture of leukocytes with cell lines prior to molecular tests [23]. On a subset of samples from the CHD group, we performed either ViroCap as described above or RT-PCR/PCR directly from buffy coat or following co-culture of buffy coat leukocytes with four mammalian cell lines: BGMK-DAF, Caco-2, HEp2, and MCR-5 (see Section 2). ViroCap was performed on 13 individual buffy coat samples from subjects with CHD who had first trimester samples available (Table S4). Herpes viruses, which have tropism for cells in buffy coat, were detected, including the Epstein–Barr Virus (EBV) and HHV-6 and HHV-7. Anelloviruses were also detected, and it has been proposed that they may replicate in mononuclear cells [24,25]. RT-PCR/PCR was performed on 29 individual buffy coat samples and media from co-cultures from subjects with CHD who had first trimester samples available (Table S5) using previously reported primer sequences for the following viruses: rhinovirus A, pegivirus, CMV, Epstein–Barr virus (EBV), HHV-6B, and adenovirus C. RT-PCR/PCR assays on these samples did not detect any viral nucleic acids.

## 4. Discussion

While there is an increasing awareness of risks associated with viral infections during pregnancy, much remains unknown regarding the consequence of viral infections on fetal development [26]. To investigate potential risk factors, we and others have examined the role of maternal infection, with a focus on “cardiotropic” viruses (i.e., viruses that have specific receptors in the fetal heart), in causing CHD [3,4]. In a preliminary investigation, we analyzed 174 CHD-affected pregnancies (vs. 268 matched healthy pregnancies) and found mothers carrying babies with CHD had a significantly higher historical incidence of upper respiratory tract and gastrointestinal infections (*p* = 0.001 and *p* = 0.004, respectively) (unpublished data, not shown). In a recent study, we investigated a case-control cohort of 122 pregnant women using ELISA and neutralization assays against coxsackievirus type B (CVB) subtypes and found significantly higher anti-CVB antibodies in pregnancies complicated by VSD (*p* = 0.001), as well as features of pulmonary atresia, suggesting the low likelihood that one virus is specifically responsible for only one type of CHD [11].

The present study was designed to determine the viral exposure of pregnant women by analyzing both the maternal plasma virome as well as by profiling the plasma for anti-CVB antibodies for which a direct association with the development of CHD has been established in mice [9]. The salient findings of our comprehensive analysis of maternal blood virome and serology throughout pregnancy are two-fold: (1) the detection of viremia can be difficult likely due to the transient nature of these episodes; and (2) exposure to CVB among CHD-affected pregnancies appears to occur at a higher degree than anticipated. We detected the presence of multiple viruses that we did not necessarily expect, highlighting the importance of identifying the potential reservoir for these viruses and expanding the examination of the virome beyond the blood. We did not detect enteroviruses in maternal plasma nor circulating leukocytes from mothers carrying babies with CHD; of note, viremia following enteroviral infection is a rare event and often limited to only 3–5 days. We tried to directly assay buffy coat nucleic acid with ViroCap to determine whether that might enhance the detection of some viruses within the blood virome. We enhanced detection of viruses with known tropism for cells in buffy coat, such as herpesviruses, but ViroCap on plasma samples actually detected a broader range of viruses. Thus, the approach used may be determined by the question being asked and if cell-associated viruses are of interest or if a broader more comprehensive view of the virome is the goal. Using a co-culture system, to enhance the detection of potential enteroviruses in the non-cytolytic form, also yielded negative results. On the other hand, the presence of anti-CVB antibodies showed that the exposure burden in women with CHD-affected pregnancies is more than twice that of the Controls. Of note, the majority of the CVB antibody-positive samples were from women with VSD-type CHD (seven out of nine women). These data are consistent with our prior animal studies; however, a definite association in humans requires further data.

One major strength was the availability of biospecimens with extensive clinical phenotypes upon which to do the analysis. While the small sample size of the CHD cohort is a limitation, there was a significant number of maternal samples obtained prior to the diagnosis of CHD, making our investigation of viral infection in plasma samples, from the first to the third trimester, the largest study of its kind. Nevertheless, a better resolution and detection rate would be obtained by focusing the collection of samples to certain weeks of heart development (week 4–10) in the first trimester. In addition, the lack of pre-pregnancy baseline antibody titers makes it difficult to attribute exposure directly during the early period of pregnancy. This potential bias, however, applies to both groups, unless there are other confounder reasons (e.g., socioeconomic status) for women with subsequent CHD-complicated pregnancies to be more exposed to enteroviruses. In addition, the presence of viral nucleic acids does not necessarily confirm infection or actively replicating viruses. Some could be transient exposures in the blood. There is also a possibility that some of the viral nucleic acid detected could have come from environmental or kit contaminants, although contaminants that occurred during sample processing are typically from bacteria, fungi, and bacteriophages [27] and would have applied to all samples as they were processed in one batch with the same reagents. Ultimately, contaminants would not have interfered with detecting CVB.

Women are more susceptible to different viral infections during pregnancy than nonpregnant women due to changes in their immunologic status [28]. Maternal viral infection in early pregnancy has been associated with various adverse outcomes for their offspring [3]. However, only single viruses or a few pathogens have been examined in past studies [3,12]. Other reservoirs (e.g., stool) which may harbor viruses in the mother remain to be examined. This is relevant since placental inflammation or dysfunction following maternal viral infection, such as that found with human papillomavirus infection [29], may also play a role in the development of CHD. Our findings also highlight that our cross-sectional analysis of viremia is not enough to comprehensively assess viral infection during critical period of pregnancy. This suggests that direct detection of viral etiologies of CHD may be better achieved by assessing samples from multiple body sites and within a relatively narrow time frame around the time of heart development in the first trimester. In conclusion, this highlights the advantages of comprehensive direct detection of viruses with ViroCap such as determining the role of specific viral strains and whether certain co-infections are more abundant in the pathogenesis of CHD. Additionally, a large-scale survey of viral infection history early in pregnancy by other means, such as phage-immunoprecipitation antibody profiling (e.g., ViroScan [30,31,32]), has the potential to reveal evidence of previously unknown/unrecognized communities of viral infection/co-infections and guide mechanistic studies on the contribution of viruses to developmental anomalies.

## Figures and Tables

**Table 1:** Cohort characteristics.

***Characteristics per subject***			
	**Controls**	**CHD**	
**No. Subject**	73	16	
**Race**	Asian: 1	White: 16	
	Black or African American: 1		
	Multiple Race: 5		
	Other: 2		
	White: 61		
	Unknown: 3		
**Hispanic or Latino (Y/N)**	Yes: 7	Yes: 0	
	No: 63	No:16	
	Unknown: 3		
***Characteristics per pregnancy***			
	**Controls**	**CHD**	
**No. Pregnancy**	88	16	***p*-value**
Maternal Age (years) *	30 ± 0.46	31 ± 1.16	0.3813
Maternal BMI *	25.65 ± 0.56	28.40 ± 2.31	0.42
Gestational Diabetes Mellitus (Y/N) *	0/87	3/13	**0.0032**
Maternal Hypoglycemia (Y/N) *	12/71	5/11	0.4729
Maternal Hyperglycemia (Y/N) *	0/83	1/14	0.1537
Alcohol Use (Y/N) *	31/55	11/5	0.7833
Tobacco Use (Y/Q/N) *	1/11/75	1/2/13	0.2885
**Child Gestation Age (days)**	271 ± 2.06	261.2 ± 6.53	0.0948
**Child Birthweight (g)**	3333 ± 73	3189 ± 247	0.6995
**Child Gender (F/M)**	38/47	8/8	0.9073
**(IgG) (U/mL)**	8.90 ± 1.40	14.43 ± 4.40	0.2925
**(IgM) (U/mL)**	7.91 ± 2.08	7.21 ± 1.92	0.9616

Maternal Age, Maternal BMI, Child Gestation Age, and Child Birthweight are shown as mean ± standard deviation. Based on available clinical data, some criteria may not add up to total number of pregnancy due to missing data. * Indicates data obtained at recruitment.

## Data Availability

Data submission to the Sequence Read Archive is in progress, and accession numbers will be provided prior to publication.

## Supplementary Materials

The following supporting information can be downloaded at: https://www.mdpi.com/article/10.3390/microorganisms11020262/s1, Table S1: Primers used for RT-PCR and PCR detection of viruses; Table S2: Summary of CVB serology assay; Table S3: Summary of ViroCap performed on plasma samples; Table S4: Summary of ViroCap performed on circulating leukocytes from the buffy coat; Table S5: Summary of PCR performed on buffy coat and co-culture media.
